# Joint Associations of Multidimensional Socioeconomic Status and Healthy Lifestyle with Prevalent Hypertension: A Large Population-Based Study in Northwest China

**DOI:** 10.3390/nu18121860

**Published:** 2026-06-09

**Authors:** Jinli Liu, Jiaomei Yang, Zhuoru Zou, Yijun Kang, Hong Yan, Shaonong Dang

**Affiliations:** 1 Department of Epidemiology and Biostatistics, School of Public Health, Xi’an Jiaotong University Health Science Center, Xi’an Jiaotong University, Xi’an 710061, China; liujinli307@xjtu.edu.cn (J.L.);; 2 Department of Endocrinology, The First Affiliated Hospital of Xi’an Jiaotong University, Xi’an Jiaotong University, Xi’an 710061, China; 3 Key Laboratory of Environment and Genes Related to Diseases (Xi’an Jiaotong University), Ministry of Education, Xi’an 710061, China

**Keywords:** hypertension, socioeconomic status, healthy lifestyle, China

## Abstract

**Background/Objectives:** The roles of healthy lifestyle and socioeconomic status (SES) in prevalent hypertension, as well as their joint patterns, remain incompletely understood. This study examined the independent, interactive, and joint associations of multidimensional SES and multiple healthy behaviors with prevalent hypertension. **Methods:** We conducted a cross-sectional analysis of baseline data from 80,218 adults enrolled in the Regional Ethnic Cohort Study in Northwest China between 2017 and 2019. The mean age of the participants was 53.9 years. SES was classified as high, middle, or low according to household per capita income, occupation, and educational attainment. Healthy lifestyle was assessed using five factors: non-smoking, moderate alcohol intake, regular physical activity, adequate coarse-grain intake, and healthy sleep patterns. Logistic regression models were used to estimate odds ratios (ORs) and 95% confidence intervals (CIs) for the associations of SES and healthy behaviors with prevalent hypertension. **Results:** Hypertension prevalence was higher in the low-SES group than in the high-SES group (45.1% vs. 28.8%). Each additional healthy behavior was associated with 16% lower odds of hypertension (OR: 0.84; 95% CI: 0.82–0.86). Within each SES stratum, participants with 0–1 healthy behaviors had the highest odds of hypertension compared with those with 4–5 healthy behaviors (high SES: OR = 1.58, 1.31–1.92; middle SES: OR = 1.62, 1.39–1.89; low SES: OR = 1.58, 1.22–2.05). A similar but weaker pattern was observed among those with 2–3 healthy behaviors (ORs ranging from 1.18 to 1.32). Among participants with 4–5 healthy behaviors, the odds of prevalent hypertension increased as SES decreased (middle SES: OR = 1.06, 95% CI: 0.97–1.16; low SES: OR = 1.29, 95% CI: 1.17–1.42; reference: high SES). This gradient was more pronounced among those with 2–3 healthy behaviors (ORs ranging from 1.24 to 1.55). **Conclusions:** Both socioeconomic disadvantage and unhealthy lifestyle were associated with higher odds of prevalent hypertension. Although adherence to a healthy lifestyle was associated with lower odds of prevalent hypertension, it did not fully attenuate the excess odds associated with socioeconomic disadvantage.

## 1. Introduction

Hypertension is a major public health challenge in China and remains a leading cause of cardiovascular and cerebrovascular disease, including stroke and myocardial infarction [1,2]. Nationally representative surveys have shown a substantial and increasing burden of hypertension among Chinese adults. The China Hypertension Survey conducted during 2012–2015 reported a weighted hypertension prevalence of 23.2% among adults aged 18 years or older, corresponding to approximately 245 million affected individuals [3]. More recent national data showed that the weighted prevalence increased to 27.5% in 2018 [4], while a 2021–2022 nationally representative survey reported a weighted prevalence of 31.6% [5]. These trends cannot be explained by biological factors alone, but are also shaped by broader social and economic conditions. Socioeconomic status (SES), as an important social determinant of health, may be linked to hypertension through multiple pathways [6]. In socioeconomically disadvantaged settings, limited education, restricted occupational opportunities, low income, and unfavorable neighborhood conditions may together reduce access to healthcare and limit health literacy [7].

Healthy lifestyle behaviors are central to both the prevention and management of hypertension [8]. Major modifiable factors include smoking, alcohol consumption, physical activity, sleep patterns, and coarse-grain intake. Coarse grains, many of which are whole-grain staple foods in the Chinese dietary context, are important sources of dietary fiber, minerals, and other bioactive components, and have been linked to improved lipid metabolism, better blood pressure control, and lower cardiovascular risk [9]. Adequate coarse-grain intake may therefore be an important part of cardiovascular health [10]. Previous studies have consistently shown that adherence to a healthy lifestyle, including non-smoking, moderate alcohol intake, regular physical activity, a balanced diet, and healthy sleep, is associated with a lower risk of hypertension [8,11,12]. However, the uptake and long-term maintenance of these behaviors often differ across socioeconomic groups, with disadvantaged populations facing greater obstacles to sustaining healthy lifestyles, including limited health literacy, constrained material resources, and less supportive living environments. Moreover, previous studies have often examined SES and lifestyle factors separately, or have treated lifestyle mainly as a confounder or mediator in analyses of socioeconomic inequalities [13]. It remains unclear whether adherence to multiple healthy behaviors is associated with lower odds of hypertension across socioeconomic groups, and whether socioeconomic disparities in hypertension persist among individuals with healthier lifestyles [14].

Northwest China is a region with substantial ethnic diversity and a distinctive hypertension profile. Local lifestyle and environmental factors may contribute to this pattern [15]. High salt and animal-fat intake may increase blood pressure through sodium retention, vascular dysfunction, weight gain, and metabolic disturbances, while cold climate may promote sympathetic activation and peripheral vasoconstriction. Relatively low physical activity may further impair cardiometabolic regulation. These factors often coexist with inequalities in healthcare access and socioeconomic development, particularly in rural and remote areas where blood pressure screening, early diagnosis, and long-term management may be limited. The clustering of environmental, social, and behavioral determinants in this region provides an important setting for examining how socioeconomic disadvantage and lifestyle behaviors jointly relate to prevalent hypertension. In particular, SES is better captured as a multidimensional construct reflecting educational, occupational, and material resources, rather than by a single indicator such as income or education alone. Therefore, using large-scale population-based data from Northwest China, we examined the distribution of healthy lifestyle behaviors across socioeconomic groups and investigated the independent, interactive, and joint associations of multidimensional SES and healthy lifestyle factors with prevalent hypertension. This study may inform context-specific and integrated strategies that address both social and behavioral determinants of hypertension.

## 2. Methods

### 2.1. Study Population

**Figure 1:**
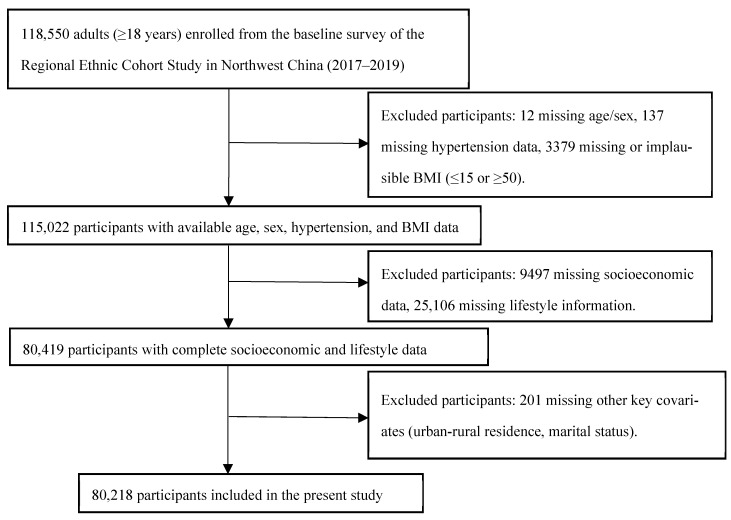
Flow diagram of participant selection in the present study.

This study was based on the Regional Ethnic Cohort Study in Northwest China (RECS) [16]. We conducted a cross-sectional analysis using baseline data from the RECS. The cohort was established under the National Key R&D Program of China as a population-based study in Northwest China, aiming to provide a large population-based research platform for this region. Between 2017 and 2019, 118,550 adults aged ≥18 years were recruited from five northwestern provinces and autonomous regions, including Shaanxi, Gansu, Qinghai, Ningxia Hui Autonomous Region, and Xinjiang Uygur Autonomous Region. Baseline information on sociodemographic characteristics, environmental and social exposures, lifestyle behaviors, medical history, and medication use was collected using standardized questionnaires administered by trained investigators. Anthropometric measurements and blood pressure measurements were conducted by trained personnel following standardized protocols. Participants with missing or implausible data on age, sex, body mass index (BMI), socioeconomic status, lifestyle factors, or other key covariates used in the regression models, including urban–rural residence and marital status, were excluded (Figure 1). After these exclusions, 80,218 participants were included in the final analysis. The study was conducted in accordance with the Declaration of Helsinki and was approved by the Human Research Ethics Committee of the Xi’an Jiaotong University Health Science Center on 11 November 2016 (approval No. XJTU2016-411). Written informed consent was obtained from all participants before data collection.

### 2.2. Assessment of Socioeconomic Status

Socioeconomic status (SES) was assessed using three indicators: educational attainment, occupational prestige, and household monthly per capita income, consistent with previous studies [17]. Each component was categorized into three levels. Educational attainment was classified as less than high school (primary school or below and junior high school), high school or equivalent (senior secondary school or secondary technical or vocational education), and college or above. Occupational prestige was defined using the Chinese occupational prestige scale, which incorporates education, income, authority, type of work unit, and occupational classification. Participants were grouped into three categories: unemployed or full-time homemakers; low-prestige occupations (score < 50), including agricultural, forestry, animal husbandry, and fishery workers, manual laborers, sales and service workers, and self-employed individuals; and high-prestige occupations (score ≥ 50), including administrative staff, managers, and professionals [18]. Household monthly per capita income was categorized according to the China MUCA-1998 criteria as <300 RMB, 300–800 RMB, and ≥800 RMB [19]. Because these thresholds were originally defined at the 1998 price level, income reported at baseline was converted to 1998 price equivalents using the consumer price index (CPI) published by the National Bureau of Statistics of China. Since the baseline survey was conducted between 2017 and 2019, we used 2018 as the reference year for the income adjustment. The cumulative CPI ratio from 1998 to 2018 was used to deflate baseline income to the 1998 price level [20]. Specifically, adjusted income was calculated as follows: Adjusted income = baseline income/cumulative CPI ratio from 1998 to 2018. Participants were then reclassified into the three income categories according to the adjusted income values.

For each SES component, scores of 1, 2, and 3 were assigned to low, medium, and high levels, respectively. These scores were summed to generate an overall SES score ranging from 3 to 9. Participants were then classified into three SES groups: low (3–4), medium (5–6), and high (≥7).

### 2.3. Assessment of Lifestyle Behavioral Factors

Based on prior evidence [21] and the available data, five modifiable lifestyle behaviors were selected to construct a composite healthy lifestyle score: smoking status, alcohol consumption, physical activity, coarse-grain intake, and sleep patterns. These behaviors were defined in the questionnaire and assessed using standardized measures; detailed definitions and scoring procedures are provided in the Supplementary Materials (Tables S1 and S2).

Participants were classified as having a healthy smoking status if they were never smokers or former smokers who had quit for at least six months for reasons unrelated to illness [22]. Lower-risk alcohol consumption was defined as non-regular drinking or light-to-moderate intake, corresponding to <30 g/day of pure alcohol for men and <15 g/day for women [22]. Physical activity was assessed using questionnaire information on the frequency, duration, and intensity of daily activities. Based on these data, total physical activity was calculated in metabolic equivalent hours per week (MET-h/week). Participants were then classified into tertiles according to the distribution of total physical activity in the analytic sample, and those in the highest tertile were considered physically active, consistent with previous large cohort studies [23]. Adequate coarse-grain intake was defined as consumption of coarse grains, including legumes, at least four times per week, consistent with previous large cohort studies [24]. Sleep patterns were assessed using a composite score based on four components: sleep duration of 7–8 h per night, absence of insomnia, no habitual snoring, and no frequent daytime sleepiness [25]. One point was assigned for each criterion met, yielding a total sleep score ranging from 0 to 4; a score of 3 or 4 was defined as a healthy sleep pattern.

For each lifestyle factor, participants received one point for a healthy behavior and zero otherwise. The overall healthy lifestyle score was calculated by summing these components, resulting in a total score ranging from 0 to 5, with higher scores indicating healthier lifestyles. Consistent with previous studies [17], participants were categorized into three groups: 0–1, 2–3, and 4–5 healthy behaviors.

### 2.4. Outcome Ascertainment

At baseline, data on blood pressure, history of hypertension, and use of antihypertensive medications were collected. Blood pressure was measured by trained personnel using a calibrated upper-arm electronic sphygmomanometer (Omron HEM-7125; Omron Healthcare Co., Ltd., Kyoto, Japan) after participants had rested in a seated position, and was recorded in millimeters of mercury (mmHg; 1 mmHg = 0.133 kPa). Blood pressure was measured three times according to the standardized study protocol, and the average of the last two measurements was used in the analysis. Hypertension was defined as systolic blood pressure ≥ 140 mmHg and/or diastolic blood pressure ≥ 90 mmHg, self-reported physician diagnosis of hypertension, or current use of antihypertensive medication [26].

### 2.5. Statistical Analysis

Baseline characteristics were summarized descriptively according to SES groups. Continuous variables were presented as means and 95% confidence intervals (CIs), and categorical variables were presented as numbers and percentages. No formal hypothesis testing or normality testing was performed for baseline characteristics, as these summaries were intended to describe the study population rather than to assess statistical differences between SES groups.

Logistic regression models were used to examine the associations of SES and healthy lifestyle scores with prevalent hypertension. Stratified analyses by sex and age were conducted to assess potential heterogeneity and robustness. All analyses were performed using R software (version 4.2.3; https://www.r-project.org/). A two-sided *p* value < 0.05 was considered statistically significant.

## 3. Results

### 3.1. Study Population Characteristics

This cross-sectional analysis included 80,218 participants from the RECS. The mean age was 53.9 years (95% CI: 53.8–54.0), and 70.4% were women (Table 1). Socioeconomic status (SES) was assessed using household per capita income, occupation, and educational attainment. Each component was scored and summed to form an overall SES index. Participants were then categorized into three groups: high (N = 17,599; 21.9%), middle (N = 42,912; 53.5%), and low SES (N = 19,707; 24.6%) (Table 1).

Compared with the high SES group, participants in the low SES group were older (mean age: 56.5 vs. 47.5 years), more often women (77.7% vs. 59.4%), and less likely to live in urban areas (6.4% vs. 60.8%). Obesity prevalence appeared to increase across decreasing SES groups, from 13.0% in the high SES group to 17.1% in the middle SES group and 20.0% in the low SES group. A similar pattern was observed for hypertension prevalence, which was highest in the low SES group (45.1%), followed by the middle SES group (40.5%) and the high SES group (28.8%) (Table 1).

A comparison of included and excluded participants is presented in Supplementary Table S3. Compared with included participants, excluded participants were younger, more likely to live in urban areas, less likely to be women, had higher educational attainment, and had a lower prevalence of hypertension. The largest differences were observed for sex (standardized mean difference, SMD = 0.69), educational attainment (maximum SMD = 0.49), urban–rural residence (SMD = 0.44), and age group (maximum SMD = 0.38), whereas the difference in BMI was minimal (SMD = 0.01). These differences suggest that potential selection bias cannot be fully excluded.

### 3.2. Association of Healthy Behaviors with Prevalent Hypertension

**Figure 2:**
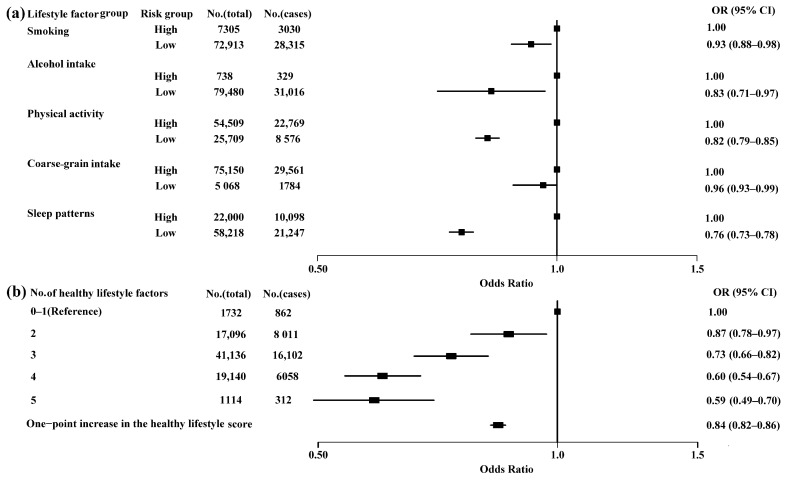
Associations of individual lifestyle factors and cumulative healthy lifestyle score with prevalent hypertension among RECS participants, 2017–2019. Odds ratios (ORs) and 95% confidence intervals (CIs) were estimated using multivariable logistic regression models adjusted for socioeconomic status, sex, age, urban–rural residence, marital status, and BMI. (**a**) Associations between individual lifestyle factors and prevalent hypertension. For each lifestyle factor, the high-risk group was used as the reference group. (**b**) Associations between the number of healthy lifestyle factors and prevalent hypertension. Participants with 0–1 healthy lifestyle factors were used as the reference group. The association per one-point increase in the healthy lifestyle score is also shown.

In multivariable models adjusted for socioeconomic status, sex, age, urban–rural residence, marital status, and BMI, all five healthy behaviors were associated with lower odds of hypertension after multivariable adjustment. Compared with their respective high-risk reference groups, the adjusted odds ratios (ORs) were 0.93 (95% CI: 0.88–0.98) for non-smoking, 0.83 (0.71–0.97) for moderate alcohol consumption, 0.82 (0.79–0.85) for regular physical activity, 0.96 (0.93–0.99) for adequate coarse-grain intake, and 0.76 (0.73–0.78) for healthy sleep patterns (Figure 2a). Stratified analyses by sex and age showed broadly consistent results. The inverse associations of moderate alcohol consumption and coarse-grain intake were mainly observed in men, whereas physical activity and healthy sleep were associated with lower odds of hypertension in both sexes. Healthy sleep was consistently associated with lower odds of hypertension across all age groups, with the strongest association observed among individuals aged 40–59 years (Table S4).

When combined into a composite lifestyle score, a clear inverse gradient was observed (Figure 2b). Compared with individuals with 0–1 healthy behaviors, the odds of hypertension decreased progressively among those with 2, 3, 4, and 5 healthy behaviors, with ORs of 0.87 (0.78–0.97), 0.73 (0.66–0.82), 0.60 (0.54–0.67), and 0.59 (0.49–0.70), respectively. Each additional healthy behavior was associated with 16% lower odds of hypertension (OR: 0.84, 0.82–0.86) (Figure 2b). Stratified analyses by sex and age showed that this inverse association was more pronounced in men and was strongest among individuals aged 40–59 years (Table S5).

### 3.3. Associations Between SES and Prevalent Hypertension Across Healthy Lifestyle Score Groups

**Figure 3:**
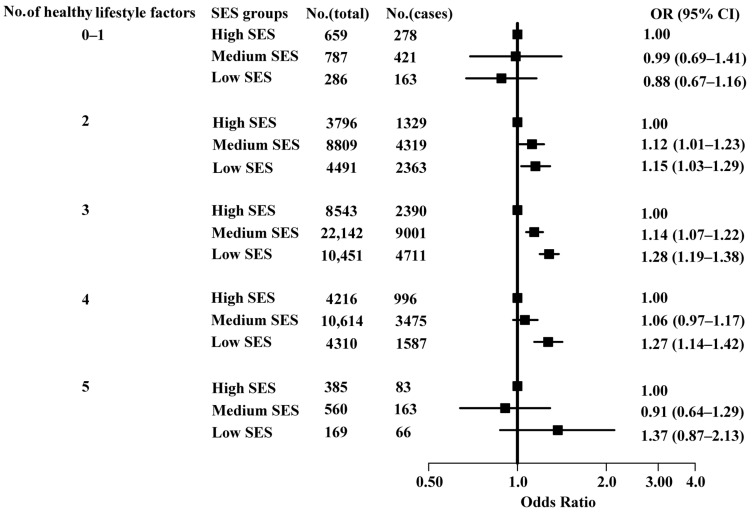
Associations between socioeconomic status (SES) and prevalent hypertension across healthy lifestyle score groups among RECS participants, 2017–2019. Odds ratios (ORs) and 95% confidence intervals (CIs) were estimated using multivariable logistic regression models adjusted for sex, age, urban–rural residence, marital status, and BMI. Within each healthy lifestyle score group, participants with high SES were used as the reference group.

Despite adherence to multiple healthy behaviors, individuals with low SES continued to show higher odds of prevalent hypertension. When stratified by the composite healthy lifestyle score, both low and middle SES groups generally showed higher odds of prevalent hypertension across most strata compared with the high SES group (Figure 3). Among participants with two healthy behaviors, the odds of prevalent hypertension were higher in the low SES (OR = 1.15, 1.03–1.29) and middle SES groups (OR = 1.12, 1.01–1.23) compared with the high SES group. A similar pattern was observed among those with three healthy behaviors, with elevated odds in the low SES (OR = 1.28, 1.19–1.38) and middle SES groups (OR = 1.14, 1.07–1.22) (Figure 3).

Among individuals with four healthy behaviors, the low SES group still had significantly higher odds of prevalent hypertension (OR = 1.27, 1.14–1.42), whereas no significant difference was observed in the middle SES group (OR = 1.06, 0.97–1.17). When all five healthy behaviors were present, no significant differences in the odds of prevalent hypertension were observed across SES groups. However, this finding should be interpreted with caution, as it may reflect limited statistical power (Figure 3).

### 3.4. Interaction and Joint Associations of SES and Lifestyle with Prevalent Hypertension

**Figure 4:**
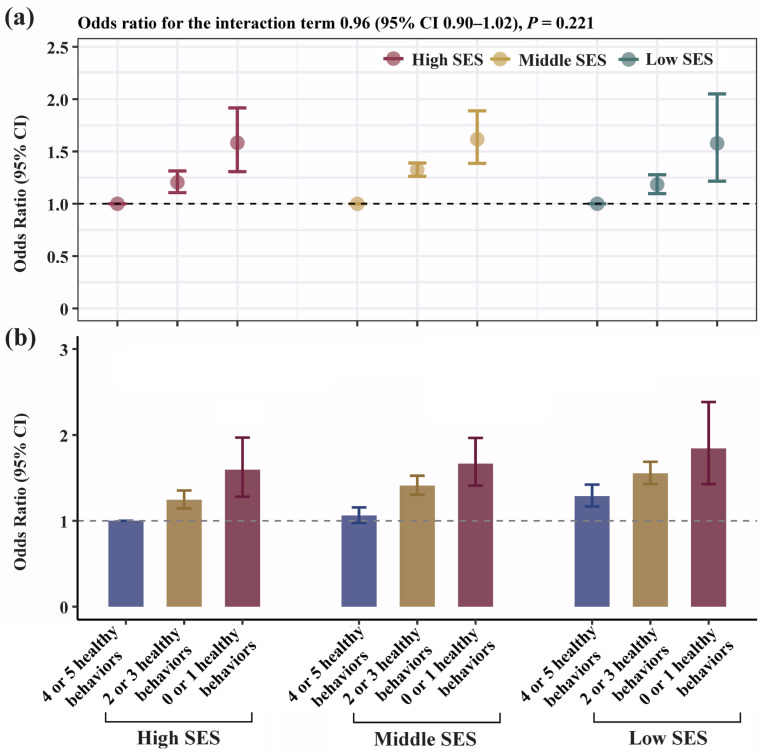
Joint associations of socioeconomic status (SES) and healthy lifestyle score with prevalent hypertension among RECS participants, 2017–2019. Odds ratios (ORs) and 95% confidence intervals (CIs) were estimated using multivariable logistic regression models adjusted for sex, age, urban–rural residence, marital status, and BMI. (**a**) Associations between healthy lifestyle score and prevalent hypertension within each SES stratum. The reference group was participants with 4–5 healthy behaviors within the same SES stratum. (**b**) Joint associations of SES and healthy lifestyle score with prevalent hypertension. The reference group was participants with high SES and 4–5 healthy behaviors. The dashed vertical line indicates the reference value of OR = 1.00.

In the multiplicative interaction analysis, no significant interaction was observed between the healthy lifestyle score and SES in relation to prevalent hypertension (OR = 0.96, 0.90–1.02). However, the healthy lifestyle score was inversely associated with the odds of prevalent hypertension within each SES stratum. Using individuals with 4–5 healthy behaviors as the reference within each SES group, those with 0–1 healthy behavior had the highest odds of prevalent hypertension across all SES levels, including high (OR = 1.58, 1.31–1.92), middle (OR = 1.62, 1.39–1.89), and low SES (OR = 1.58, 1.22–2.05). A similar but weaker pattern was observed among those with 2–3 healthy behaviors, with ORs ranging from 1.18 to 1.32 across SES groups (Figure 4a).

In the joint analysis, the odds of prevalent hypertension increased as SES decreased and the number of healthy behaviors declined. Across all levels of healthy behaviors, individuals with lower SES consistently had higher odds of prevalent hypertension than those with higher SES. For example, among participants with 4–5 healthy behaviors, the odds increased with decreasing SES: 1.00 (reference) in the high SES group, 1.06 (0.97–1.16) in the middle SES group, and 1.29 (1.17–1.42) in the low SES group, indicating that differences in the odds of prevalent hypertension persisted even among those with the healthiest behaviors (Figure 4b). A similar pattern was observed among those with 2–3 healthy behaviors, with a more pronounced increase in the odds of prevalent hypertension across SES groups (ORs ranging from 1.24 to 1.55) (Figure 4b). Overall, these findings suggest that although a healthy lifestyle was associated with lower odds of prevalent hypertension, it did not fully eliminate the excess odds associated with lower SES.

## 4. Discussion

Drawing on large-scale data from the RECS, this study examined the independent, stratified, and joint associations of SES and cumulative healthy lifestyle behaviors with prevalent hypertension. Three main findings can be highlighted. First, the odds of prevalent hypertension showed a clear socioeconomic gradient, increasing steadily as SES declined. Second, all five healthy behaviors were associated with lower odds of prevalent hypertension and showed a clear cumulative effect. As the number of healthy behaviors increased, the odds of prevalent hypertension decreased in a graded manner. Each additional healthy behavior was associated with approximately 16% lower odds of hypertension, suggesting a robust graded association. Third, although adherence to a comprehensive healthy lifestyle was associated with substantially lower odds of prevalent hypertension, it did not fully remove the excess odds associated with socioeconomic disadvantage. Given the high proportion of women in the analytic sample, sex-stratified analyses were conducted to further examine the robustness of the observed associations. The inverse association between the composite healthy lifestyle score and prevalent hypertension was generally observed in both men and women. Some individual lifestyle factors showed sex-specific patterns. For example, the associations of alcohol consumption and coarse-grain intake with prevalent hypertension were more evident among men, whereas physical activity and healthy sleep were associated with lower odds of hypertension in both sexes. These findings suggest that the association between an overall healthier lifestyle and lower odds of prevalent hypertension was not driven solely by the female-dominant sample.

Hypertension burden increased with decreasing SES and was substantially higher among individuals with lower SES. In our study, the prevalence of hypertension reached 45.1% in the low-SES group, compared with 28.8% in the high-SES group, indicating a marked disparity in cardiovascular health. This pattern is consistent with global epidemiological evidence and with findings from other regions in China [3,27]. Individuals with lower SES are more likely to face job insecurity, financial strain, and limited health literacy. These conditions may affect the hypothalamic–pituitary–adrenal axis and the autonomic nervous system, increasing allostatic load and contributing to sustained elevation in blood pressure [28,29,30]. In the particular socio-ecological setting of Northwest China, these associations may be further shaped by structural constraints. People with lower SES often have poorer access to high-quality healthcare [31], long-term exposure to high-salt dietary patterns, such as those reported in Shaanxi Province [32], and fewer opportunities for physical activity [33]. Together, these structural and behavioral disadvantages may contribute to unfavorable blood pressure profiles and persistent socioeconomic differences in hypertension, making it more difficult to reduce health inequalities in this population.

Our findings further suggest a cumulative inverse association between healthy lifestyle and prevalent hypertension. The odds of hypertension decreased progressively with an increasing number of healthy behaviors, with each additional behavior associated with approximately 16% lower odds of hypertension. This pattern is biologically plausible, as multiple healthy behaviors may be linked to blood pressure through complementary mechanisms, including improved endothelial function, reduced systemic inflammation, better metabolic control, and maintenance of autonomic and neuroendocrine homeostasis [34]. Lower-risk alcohol consumption, defined in our study as non-regular drinking or light-to-moderate alcohol intake, was associated with lower odds of hypertension. Excessive alcohol consumption is a well-established risk factor for hypertension and may increase blood pressure through sympathetic activation, impaired baroreflex sensitivity, oxidative stress, endothelial dysfunction, and activation of the renin–angiotensin–aldosterone system [35]. Therefore, our findings should not be interpreted as recommending alcohol consumption, but rather as supporting the prevention of heavy and harmful drinking as part of an overall healthy lifestyle strategy. Physical activity and coarse-grain intake may improve endothelial function, increase nitric oxide bioavailability, and reduce systemic inflammation, thereby contributing to blood pressure regulation [36,37,38]. Sleep health may be linked to hypertension through autonomic, circadian, inflammatory, and vascular pathways [39]. Adequate and regular sleep may help maintain the normal circadian rhythm of blood pressure, including nocturnal blood pressure dipping [39]. By contrast, short sleep duration, insomnia, habitual snoring, and daytime sleepiness may reflect sleep fragmentation or sleep-disordered breathing, which can increase sympathetic activity, promote vasoconstriction, sodium retention, and activation of the renin–angiotensin–aldosterone system [39,40]. Poor sleep may also increase oxidative stress and low-grade inflammation, thereby contributing to sustained blood pressure elevation [41,42]. Smoking cessation has been linked to improved endothelial function and reduced oxidative stress and vascular injury, thereby helping to prevent atherogenic changes and lower blood pressure [43]. By incorporating factors such as sleep quality and coarse-grain intake, which are less often included in conventional lifestyle assessments, into a composite lifestyle score, our analysis further supports the relevance of multidimensional lifestyle assessment for cardiometabolic health research [44,45]. These findings provide evidence from an East Asian population and may help inform population-level strategies for hypertension control and health equity.

A key finding of this study lies in the joint analysis of SES and lifestyle behaviors. Although healthy behaviors were associated with substantially lower odds of hypertension, they did not fully eliminate the higher odds of prevalent hypertension associated with low SES. This pattern suggests that SES and lifestyle do not appear to substitute for one another, but rather show a cumulative association with the odds of prevalent hypertension [28]. From a mechanistic perspective, healthy behaviors may be linked to blood pressure through modifiable pathways, including sympathetic activity, endothelial function, and systemic inflammation [30,43,46,47]. In contrast, low SES may reflect more persistent structural and psychosocial disadvantages, including limited access to high-quality healthcare, adverse living and working conditions, and cumulative social stress [6]. These factors may contribute to unfavorable blood pressure profiles and help explain why socioeconomic differences in hypertension persist even among individuals reporting healthier lifestyles. Although previous studies have reported associations of socioeconomic disadvantage and unhealthy lifestyle with hypertension or cardiovascular health outcomes [14,17,19], our study extends existing knowledge by examining their joint pattern in a large population from Northwest China. The finding that a healthier lifestyle was associated with lower odds of hypertension but did not fully eliminate the excess odds associated with low SES suggests that behavioral interventions alone may be insufficient to reduce socioeconomic inequalities in hypertension. Prevention strategies should therefore combine lifestyle promotion with broader structural measures that improve health literacy, healthcare access, and supportive environments for disadvantaged populations.

This study has several methodological strengths and important public health implications. Using baseline data from the large-scale RECS, we examined hypertension within a distinctive ecological and socio-cultural context characterized by diverse ecological environments, including high-altitude areas, multi-ethnic populations, and traditional high-salt and high-fat dietary patterns. At the same time, several limitations should be acknowledged. First, the temporal relationship between SES, lifestyle behaviors, and hypertension status cannot be firmly established in this cross-sectional analysis. It is possible that some individuals adopted healthier behaviors after being diagnosed with hypertension, which may have led to an underestimation of the associations between unhealthy lifestyles and hypertension. Prospective cohort studies are needed to address this issue. Second, the composite SES index may not fully capture the complexity of socioeconomic disadvantage. Income, education, and occupation represent different dimensions of socioeconomic position, but their relative importance may vary across populations and may also be correlated with age, sex, and urban–rural residence. Therefore, the SES score should be interpreted as a pragmatic summary measure of socioeconomic position in this cohort rather than as a comprehensive deprivation index. Third, residual confounding should also be considered. Although we adjusted for several major demographic and anthropometric factors, some potentially relevant variables were not directly measured or were not available for inclusion in the main models, including sodium intake, psychosocial stress, diabetes, dyslipidemia, chronic kidney disease, and detailed information on specific antihypertensive drug classes. These factors may be associated with socioeconomic position, lifestyle behaviors, and hypertension, and may therefore have influenced the observed associations. Fourth, a substantial number of participants were excluded because of missing or implausible data on key variables. Although these exclusions were necessary to ensure complete information on the outcome, main exposures, and covariates used in the multivariable models, excluded participants differed from included participants in several baseline characteristics, including sex, educational attainment, urban–rural residence, and age group. Therefore, potential selection bias cannot be fully excluded, and the findings should be interpreted with caution. Finally, although current antihypertensive medication use was considered in the definition of hypertension, detailed information on specific antihypertensive drug classes was not incorporated into the present analysis. Future studies with more detailed treatment information may further examine how medication patterns influence hypertension control across socioeconomic groups.

## 5. Conclusions

In summary, findings from this large population-based study in Northwest China suggest that both socioeconomic disadvantage and unhealthy lifestyle behaviors were associated with higher odds of prevalent hypertension. Although adherence to a comprehensive healthy lifestyle was associated with substantially lower odds of prevalent hypertension, it did not fully attenuate the excess odds associated with socioeconomic disadvantage. Future hypertension control strategies may need to combine lifestyle promotion with structural support for socioeconomically disadvantaged populations.

## Figures and Tables

**Table 1:** Baseline characteristics of RECS participants according to socioeconomic status (SES) at baseline (2017–2019).

Characteristics	Total Population N = 80,218	High SES N = 17,599	Medium SES N = 42,912	Low SES N = 19,707
**Age, year,** **mean (95% CI)**	53.9 (53.8–54.0)	47.5 (47.3–47.7)	55.2 (55.1–55.3)	56.5 (56.4–56.6)
**Age group, year, %**				
<40	8665 (10.8)	4693 (26.7)	2618 (6.1)	1354 (6.9)
40–59	44,658 (55.7)	9949 (56.5)	24,959 (58.2)	9750 (49.5)
≥60	26,895 (33.5)	2957 (16.8)	15,335 (35.7)	8603 (43.6)
**Urban area, %**	18,482 (23.0)	10,699 (60.8)	6515 (15.2)	1268 (6.4)
**Women, %**	56,448 (70.4)	10,446 (59.4)	30,684 (71.5)	15,318 (77.7)
**Per capita household income**				
High	32,015 (39.9)	16,512 (93.8)	15,503 (36.1)	0
Medium	31,562 (39.4)	866 (4.9)	24,847 (57.9)	5849 (29.7)
Low	16,641 (20.7)	221 (1.3)	2562 (6.0)	13,858 (70.3)
**Education**				
College or above	8465 (10.6)	8106 (46.1)	359 (0.8)	0
High school or equivalent	28,658 (35.7)	9407 (53.5)	17,913 (41.7)	1338 (6.8)
Less than high school	43,095 (53.7)	86 (0.5)	24,640 (57.4)	18,369 (93.2)
**Occupation**				
Higher prestige score	6831 (8.5)	6684 (38.0)	147 (0.3)	0
Lower prestige score	49,918 (62.2)	10,082 (57.3)	30,509 (71.1)	9327 (47.3)
Unemployed and full-time homemakers	23,469 (29.3)	833 (4.7)	12,256 (28.6)	10,380 (52.7)
**BMI, kg/m^2^,** **mean (95% CI)**	24.7 (24.6–24.8)	24.2(24.1–24.3)	24.7 (24.6–24.8)	24.9 (24.8–25.0)
**BMI group, kg/m^2^, %**				
<18.5	2324 (2.9)	513 (2.9)	1109 (2.6)	702 (3.6)
18.5–23.9	34,194 (42.6)	8274 (47.0)	17,826 (41.5)	8094 (41.1)
24.0–27.9	30,158 (37.6)	6525 (37.1)	16,655 (38.8)	6978 (35.4)
≥28.0	13,542 (16.9)	2287 (13.0)	7322 (17.1)	3933 (20.0)
**Hypertension, %**	31,345 (39.1)	5076 (28.8)	17,379 (40.5)	8890 (45.1)

Note: Values are presented as N (%) unless otherwise indicated. Age and BMI are presented as mean (95% CI).

## Data Availability

The datasets are not available for download to protect the confidentiality of the participants. The data are held at the School of Public Health, Xi’an Jiaotong University Health Science Center. If anyone wants to obtain data from this study, please contact the corresponding author Shaonong Dang.

## Supplementary Materials

The following supporting information can be downloaded at: https://www.mdpi.com/article/10.3390/nu18121860/s1, Supplementary Methods; Table S1: Questionnaire items for smoking, alcohol consumption, and sleep patterns; Table S2: Intensity categories and MET values for different types of physical activities; Table S3: Comparison of baseline characteristics between included and excluded participants; Table S4: Multivariable-adjusted odds ratios (ORs) for prevalent hypertension associated with individual lifestyle factors, stratified by sex and age group; Table S5: Multivariable-adjusted odds ratios (ORs) for prevalent hypertension according to the cumulative number of healthy lifestyle factors, stratified by sex and age group. References [48,49,50] are cited in Supplementary Materials.

## Abbreviations

BMI	Body mass index
RECS	Regional Ethnic Cohort Study in Northwest China
OR	Odds ratio
SES	Socioeconomic status
