# Supplementary material for: Joint Associations of Multidimensional Socioeconomic Status and Healthy Lifestyle with Prevalent Hypertension: A Large Population-Based Study in Northwest China
Source: Nutrients. 2026 Jun 9;18(12):1860. doi: 10.3390/nu18121860 (PMC13305019; doi:10.3390/nu18121860)
Supplement: Supplementary file 1 [file nutrients-18-01860-s001.zip › nutrients-4327141-supplementary.pdf]

Supplementary Material：

Joint Associations of Multidimensional Socioeconomic Status and Healthy Lifestyle with Prevalent Hypertension: A Large Population-Based Study in Northwest China

This supplementary material provides details on the definitions of lifestyle factors, their calculation methods, and the related results.

Contents

1. Supplementary Methods and Supplementary Tables……2

1.1 Assessment of lifestyle factors……2

1.2 Smoking, alcohol, and sleep patterns……2

1.3 Assessment of coarse-grain intake……5

1.4 Assessment of alcohol consumption……5

1.5 Quantification of alcohol intake……5

1.6 Assessment of physical activity……6

Table S1. Questionnaire items for smoking, alcohol consumption, and sleep patterns……3

Table S2. Intensity categories and MET values for different types of physical activities……8

Table S3. Comparison of baseline characteristics between included and excluded participants……10

Table S4. Multivariable-adjusted odds ratios (ORs) for prevalent hypertension associated with individual lifestyle factors, stratified by sex and age group……11

Table S5. Multivariable-adjusted odds ratios (ORs) for prevalent hypertension according to the cumulative number of healthy lifestyle factors, stratified by sex and age group……12

1. Supplementary Methods and Supplementary Tables

1.1 Assessment of lifestyle factors

Lifestyle factors were assessed through a combination of questionnaire-based measures, including smoking, alcohol consumption, sleep patterns, physical activity, and diet. Table S1 summarizes the questionnaire items and corresponding response options for smoking, alcohol consumption, and sleep behaviors.

1.2 Smoking, alcohol, and sleep patterns

Smoking behavior was evaluated through questions on current and past smoking frequency, duration since cessation, and reasons for quitting. Alcohol consumption was assessed by self-reported frequency over the past 12 months, with participants categorized into five groups: (1) never or almost never, (2) only on special occasions, (3) only during certain months, (4) throughout the year but less than once per week, and (5) at least once per week. Among regular drinkers, additional information was collected on drinking frequency, beverage type, and quantity. Alcohol intake was quantified in grams of pure alcohol per week, based on standard units for beer, rice wine, wine/fruit wine, and spirits with different alcohol concentrations, combined with the reported frequency.

Sleep patterns were assessed in terms of typical sleep duration, insomnia symptoms (difficulty falling asleep, early awakening), snoring, and excessive daytime sleepiness. Participants reported the frequency of these occurrences over the past month, enabling categorization of sleep behaviors relevant to health outcomes. Detailed information is provided in Table S1.

Table S1. Questionnaire items for smoking, alcohol consumption, and sleep patterns

Healthy lifestyle factors	Questions	Response options
**Smoking**	a) How often do you smoke tobacco now?	1) Do not smoke now<br>2) Only occasionally<br>3) Yes, on most days<br>4) Yes, daily or almost every day
	b) In the past, how frequently did you smoke?	1) Did not smoke<br>2) Smoked only occasionally<br>3) Smoked on most days<br>4) Smoked daily or almost every day
	c) How many years ago did you last stop smoking regularly?	_____years ______months
	d) What was your main reason for stopping?	1) Physical illness that you already had<br>2) Health concerns (about future illness)<br>3) Money<br>4) Family against<br>5) Medical advice<br>6) Other
**Alcohol intake**	a) During the past 12 months, how often did you drink any alcohol?	1) Never or almost never<br>2) Only on special occasions<br>3) Only during certain months<br>4) Throughout the year but less than once per week<br>5) At least once per week

	b) During the past 12 months, on how many days did you drink alcohol in a typical week?	1) 1-2 days/week<br>2) 3-5 days/week<br>3) Daily or almost every day
	c) On days when you drink, how much alcohol do you usually drink in a day?<br><br>**Alcohol type** / **On a typical day (choose one)** / **On a special day when you drink a lot** / **Last time when you drank**<br>Beer (500mL): ______Bottle / _____Bottle / _____Bottle<br>Spirits (≥40% alcohol): ______liang / ______liang / ______liang<br>Spirits (<40% alcohol): ______liang / ______liang / ______liang<br>Wine/fruit wine: ______liang / ______liang / ______liang<br>Rice wine: ______liang / ______liang / ______liang	
**Sleep patterns**		
Sleep duration	How many hours do you typically sleep per night?	______hours
Insomnia	During the past month, did you take >30 minutes to fall asleep after going to bed or waking up in the middle of the night for >3 days per week?	1) Yes<br>2) No
	During the past month, did you wake up early and were not able to go back to sleep for >3 days per week?	1) Yes<br>2) No
Snoring	Do you snore during sleep?	1) Yes, always<br>2) Yes, sometimes<br>3) No/don't know
Excessive daytime sleepiness	During the past month, because of poor sleep, did you have difficulty staying alert while at work, eating or meeting people during daytime for >3 days per week?	1) Yes<br>2) No

1.3 Assessment of coarse-grain intake

In the Chinese dietary context, coarse grains generally refer to staple grains other than refined rice, wheat, and their products, such as white rice, noodles, and white bread, and may also include common legumes depending on dietary classification. In the China Kadoorie Biobank (CKB) study, staple foods other than rice and wheat products were classified as "other staple foods" [1]. In the present study, a simplified food frequency questionnaire (FFQ) was used to assess participants' intake of various food groups. In the Chinese version of the questionnaire, this dietary item was labeled as "Za Liang", referring to commonly consumed coarse grains in China. This classification is broadly consistent with dietary assessment approaches used in previous large-scale Chinese population studies [2,3]. To improve clarity and align the exposure definition with the questionnaire, we classified both "Za Liang" and legumes as coarse grains in this study.

Participants were asked to recall their frequency of coarse-grain intake, including legumes, over the past 12 months and select from five response options: (1) daily; (2) 4–6 times per week; (3) 1–3 times per week; (4) 1–3 times per month; and (5) rarely or never. Participants reporting consumption of coarse grains, including legumes, at least four times per week were classified as having adequate coarse-grain intake.

This standardized dietary assessment approach enabled us to examine the association between coarse-grain intake and prevalent hypertension while facilitating comparison with previous large-scale Chinese population studies that collected similar dietary information [1–3].

1.4 Assessment of alcohol consumption

Participants were asked about their alcohol consumption over the past year and categorized into five groups: (1) never or almost never drank alcohol; (2) drank only on special occasions; (3) drank only during certain months; (4) drank throughout the year but less than once per week; and (5) drank at least once per week throughout the year.

Among regular drinkers, additional information on drinking frequency was collected: 1–2 days/week, 3–5 days/week, or daily/almost daily. Information on types of alcoholic

beverages consumed was also obtained, including beer, rice wine (huangjiu/mijiu), wine or fruit wine, low-alcohol-content liquor (<40% alcohol by volume), and high-alcohol-content liquor (≥40% alcohol by volume).

1.5 Quantification of alcohol intake

Daily alcohol intake was estimated based on standard units. Beer consumption was recorded in bottles (500 mL per bottle), while other alcoholic beverages were recorded in 50 g units. Average weekly alcohol consumption was calculated based on drinking frequency and then converted into grams of pure alcohol per week (g/week). Drinking frequencies of 1–2 days/week, 3–5 days/week, and daily/almost daily were assigned weights of 1.5, 4, and 6.5 days per week, respectively.

The alcohol content of beer was assumed to be 4%, corresponding to 16 g of pure alcohol per bottle (0.04 × 500 × 0.8). For other beverages, one unit (50 g) was assumed: rice wine (11.8% alcohol, 5.9 g pure alcohol), wine/fruit wine (9.5%, 4.75 g), high-alcohol liquor (41.8%, 20.9 g), and low-alcohol liquor (30%, 15 g).

1.6 Assessment of physical activity

Physical activity over the past year was assessed, including work-related activities, commuting mode and duration, leisure-time physical exercise, and household activities. The questionnaire differed slightly between agricultural (farming, forestry, animal husbandry, and fishery) and non-agricultural workers.

Metabolic equivalent of task (MET) values for different types of physical activities were obtained from standard references (Table S2). Based on questionnaire responses and the compendium of physical activities developed by Ainsworth et al. [4], total physical activity for each participant was calculated as MET-hours per week (MET-h/week) using the following formula:

$$\text{Total MET-hours/week} = \sum_i MET_i \times \mathrm{T}_i \times \mathrm{D}_i$$

where MET_i represents the metabolic equivalent of the *i*-th activity, T_i represents the average duration per day spent in that activity, and D_i represents the number of days per week spent in that activity.

Each healthy lifestyle factor was assigned one point if the criterion was met and zero points otherwise. The five components were summed to generate a healthy lifestyle score ranging from 0 to 5. Participants were categorized into 0–1, 2–3, and 4–5 healthy lifestyle factors according to the total score.

Table S2. Intensity categories and MET values for different types of physical activities

Category	Activity Type	Intensity	MET	Code/Reference
Work	Mainly sedentary	Light	1.8	Mean (11580, 11585, 11590)
	Mainly standing	Moderate	3.8	Mean (11610, 11630)
	General physical labor	Moderate	4.3	11477
	Heavy physical labor	Vigorous	6.5	11477
Agricultural work	Manual labor during busy farming season	Vigorous	6.3	Mean (11145, 11146)
	Semi-mechanized work during slack season	Moderate	3.4	Mean (11146, 11147)
	Mechanized work during slack season	Moderate	2.4	Mean (11147, 11170)
	Light work during slack season	Light	2.0	11147
	Intensive labor during busy season	Vigorous	7.8	11145
Transportation	Walking	Moderate	4.0	17170
	Cycling	Vigorous	6.8	01011
	Motorcycling	Moderate	3.5	16030
	Private car / Taxi	Light	1.9	Mean (16010, 16015)
	Public transport (bus, subway, ferry)	Light	1.7	16016
Household Activities	Household chores	Light	2.8	Mean (05030[a], 05040[a], 05035, 05055, 05070, 05090[a], 05092[a], 05184, 05197, 05200)
Physical Exercise	Vigorous exercise	Vigorous	8.0	Mean (PAD615, PAD660)
	Tai Chi / Qigong / Walking / Yoga	Moderate	3.3	Mean (15670, 17160)
	Running / Aerobics	Vigorous	7.4	Mean (12020, 12150, 03015)
	Ball sports	Moderate	5.5	Mean (15020[a], 15030[a], 15055, 15080, 15090, 15255, 15605[a], 15610, 15652, 15660, 15675, 15710[a], 15711[a])
Physical exercise	Swimming	Vigorous	7.2	Mean (18230, 18240, 18310)
	Brisk walking / Yangko dance / Square dancing	Moderate	4.2	Mean (03025, 15300, 17200)

	Other activities (e.g., hiking, cycling, rope skipping, shuttlecock kicking)	Moderate	5.9	15310, 15425[a], 15430[a], 15537, 15550[b], 15551[b], 15552[b], 15580, 15590, 15730, 15732[b], 15733[b], 15734[b], 19030
Sedentary Activities	Watching television	Light	1.2	Mean (07010, 07020)
	Using a mobile phone / iPad	Light	1.7	Mean (09050, 09055)
	Playing chess/cards/mahjong	Light	1.5	Mean (09000, 09010, 09013)
	Reading books/newspapers	Light	1.5	Mean (09030, 09070, 07070)
	Computer use / writing / document processing	Light	1.5	Mean (09015, 09040, 09045, 09050[a], 09055[a], 09060, 09075, 09070, 07050, 07070)

Notes:

- **MET**: Metabolic Equivalent of Task.
- [a]: Assigned a weight of 1/2 when calculating the average MET (two items represent the same activity type).
- [b]: Assigned a weight of 1/3 when calculating the average MET (three items represent the same activity type).
- Data source: Activity items were adapted from the NHANES physical activity questionnaire, and MET values were assigned according to the 2011 Compendium of Physical Activities by Ainsworth et al. [4]. (https://wwwn.cdc.gov/Nchs/Data/Nhanes/Public/2011/DataFiles/PAQ_G.htm)

Table S3. Comparison of baseline characteristics between included and excluded participants

Characteristics	Included participants N=80,218	Excluded participants N=38,332	SMD
**Age, years, mean (95% CI)**	53.9 (53.8–54.0)	49.4 (49.3–49.5)	0.36
**Age group, years, %**			0.38
<40	8665 (10.8)	9670 (25.2)	-
40–59	44,658 (55.7)	19,140 (49.9)	-
≥60	26,895 (33.5)	9521 (24.8)	-
**Urban area, %**	18,482 (23.0)	16,593 (43.3)	0.44
**Women, %**	56,448 (70.4)	14,443 (37.7)	0.69
**Per capita household income**			0.30
High	32,015 (39.9)	14,251 (37.2)	-
Medium	31,562 (39.4)	9753(25.4)	-
Low	16,641(20.7)	4910 (12.8)	-
**Education**			0.49
College or above	8465 (10.6)	11,416 (29.8)	-
High school or equivalent	28,658 (35.7)	13,776 (35.9)	-
Less than high school	43,095 (53.7)	12,672 (33.1)	-
**Occupation**			0.33
Higher prestige score	6831 (8.5)	7600 (19.8)	-
Lower prestige score	49,918 (62.2)	21,460 (56.0)	-
Unemployed and full-time homemakers	23,469 (29.3)	7847 (20.5)	-
**BMI, kg/m^2, mean (95% CI)**	24.7 (24.6–24.8)	24.6 (24.4–24.8)	0.01
**BMI group, kg/m^2, %**			0.11
<18.5	2324 (2.9)	1324 (3.5)	-
18.5–23.9	34,194 (42.6)	16,064 (41.9)	-
24.0–27.9	30,158 (37.6)	12,706 (33.1)	-
≥28.0	13,542 (16.9)	5025 (13.1)	-
**Hypertension, %**	31,345 (39.1)	11,910 (32.6)	0.14

Note: Values are presented as mean (95% CI) or n (%). Percentages were calculated among participants with available data for each characteristic, because excluded participants had missing information on one or more key variables. Therefore, the number of participants with available data may vary across characteristics among excluded participants. SMD, standardized mean difference. SMDs are unitless measures used to quantify the magnitude of differences between included and excluded participants. For categorical variables with more than two categories, the maximum category-specific SMD is presented.

Table S4. Multivariable-adjusted odds ratios (ORs) for prevalent hypertension associated with individual lifestyle factors, stratified by sex and age group

Group	Lifestyle factor	High risk		Low risk		ORs (95% CI)
		No. (total)	No. (cases)	No. (total)	No. (cases)	
Sex	**Men**					
	Smoking	6376	2701	17,394	7417	0.95 (0.89–1.00)
	Alcohol intake	545	258	23,225	9860	0.78 (0.65–0.94)
	Physical activity	15,278	7028	8492	3090	0.78 (0.73–0.83)
	Coarse-grain intake	22,488	9645	1282	473	0.86 (0.76–0.97)
	Sleep patterns	6647	3196	17,123	6922	0.78 (0.74–0.83)
	**Women**					
	Smoking	929	329	55,519	20,898	1.00 (0.87–1.16)
	Alcohol intake	193	71	56,255	21,156	1.14 (0.84–1.56)
	Physical activity	39,213	15,741	17,217	5486	0.84 (0.81–0.88)
	Coarse-grain intake	52,662	19,916	3786	1311	0.99 (0.93–1.08)
	Sleep patterns	15,353	6902	41,095	14,325	0.81 (0.78–0.84)
Age groups	**<40 years**					
	Smoking	974	157	7691	925	0.87 (0.71–1.08)
	Alcohol intake	92	23	8573	1059	0.64 (0.39–1.09)
	Physical activity	5282	649	3383	433	0.94 (0.82–1.08)
	Coarse-grain intake	8023	1016	642	66	1.05 (0.79–1.38)
	Sleep patterns	1677	257	6988	825	0.79 (0.67–0.93)
	**40–59 years**					
	Smoking	3596	1386	41,062	14,324	0.89 (0.82–0.96)
	Alcohol intake	417	191	44,241	15,519	0.73 (0.60–0.90)
	Physical activity	27,555	10,110	17,103	5600	0.82 (0.79–0.85)
	Coarse-grain intake	41,846	14,750	2812	960	1.10 (0.96–1.25)
	Sleep patterns	10,900	4393	33,758	11,317	0.76 (0.73–0.80)
	**≥60 years**					
	Smoking	2735	1487	24,160	13,066	0.98 (0.89–1.07)
	Alcohol intake	229	115	26,666	14,438	1.14 (0.88–1.49)
	Physical activity	21,672	12,010	5223	2543	0.79 (0.74–0.84)
	Coarse-grain intake	25,281	13,795	1614	758	0.77 (0.69–0.86)
	Sleep patterns	9423	5448	17,472	9105	0.84 (0.80–0.88)

Note: ORs and 95% confidence intervals (CIs) were estimated using multivariable logistic regression models. For each lifestyle factor, the high-risk group was used as the reference category. Models were adjusted for socioeconomic status, sex, age, urban–rural residence, marital status, and body mass index, except that the stratification variable was not included as a covariate in the corresponding stratified model.

Table S5. Multivariable-adjusted odds ratios (ORs) for prevalent hypertension according to the cumulative number of healthy lifestyle factors, stratified by sex and age group

Group	Number of healthy lifestyle factors	No. (total)	No. (cases)	ORs (95% CI)
**Sex**	**Men**			
	0-1 (Reference)	1534	776	1.00
	2	6157	2900	0.87 (0.78–0.98)
	3	10,798	4608	0.75 (0.67–0.85)
	4	5059	1766	0.59 (0.53–0.67)
	5	222	68	0.55 (0.40–0.75)
	Per one-point increase in healthy lifestyle score	-	-	0.84 (0.82–0.87)
	**Women**			
	0-1 (Reference)	198	86	1.00
	2	10,939	5111	0.96 (0.70–1.31)
	3	30,338	11,494	0.82 (0.60–1.11)
	4	14,081	4292	0.68 (0.5–0.93)
	5	892	244	0.68 (0.48–0.96)
	Per one-point increase in healthy lifestyle score	-	-	0.85 (0.83–0.88)
**Age groups**	**<40 years**			
	0-1 (Reference)	209	44	1.00
	2	1301	190	0.80 (0.54–1.19)
	3	4328	520	0.69 (0.48–1.02)
	4	2641	313	0.65 (0.44–0.96)
	5	186	15	0.55 (0.28–1.03)
	Per one-point increase in healthy lifestyle score	-	-	0.89 (0.82–0.97)
	**40-59 years**			
	0-1 (Reference)	677	317	1.00
	2	7810	3155	0.76 (0.64–0.90)
	3	22,709	8056	0.64 (0.54–0.75)
	4	12,716	3956	0.52 (0.44–0.61)
	5	746	226	0.56 (0.45–0.70)
	Per one-point increase in healthy lifestyle score	-	-	0.83 (0.81–0.86)
	**≥60 years**			
	0-1 (Reference)	846	501	1.00
	2	7985	4666	0.96 (0.83–1.11)
	3	14,099	7526	0.82 (0.70–0.95)
	4	3783	1789	0.66 (0.56–0.77)
	5	182	71	0.49 (0.35–0.69)
	Per one-point increase in healthy lifestyle score	-	-	0.84 (0.82–0.87)

Note: ORs and 95% confidence intervals (CIs) were estimated using multivariable logistic regression models. Participants with 0–1 healthy lifestyle factors were used as the reference group. Models were adjusted for socioeconomic status, sex, age, urban–rural residence, marital status, and body mass index, except that the stratification variable was not included as a covariate in the corresponding stratified model.

Reference:

1. Du, H.; Li, L.; Bennett, D.; Guo, Y.; Key, T.J.; Bian, Z.; Sherliker, P.; Gao, H.; Chen, Y.; Yang, L.; et al. Fresh Fruit Consumption and Major Cardiovascular Disease in China. *New Engl. J. Med*. **2016**, 374, 1332–1343. https://doi.org/10.1056/nejmoa1501451.
2. Li, C.; Liu, Y.; Shi, G.; Mi, B.; Zeng, L.; Li, Q.; Shen, Y.; Zhao, Y.; Pei, L.; Kang, Y.; et al. Cohort Profile: Regional Ethnic Cohort Study in Northwest China. *Int. J. Epidemiol*. **2022**, 51, e18–e26.
3. Li, L.M.; Lv, J.; Guo, Y.; Collins, R.; Chen, J.S.; Peto, R.; Wu, F.; Chen, Z.M.; China Kadoorie Biobank (CKB) Collaborative Group. The China Kadoorie Biobank: Related methodology and baseline characteristics of the participants. *Zhonghua Liu Xing Bing Xue Za Zhi.* **2012**, 33, 249–255. (In Chinese). https://pubmed.ncbi.nlm.nih.gov/22613372/.
4. Ainsworth, B.E.; Haskell, W.L.; Herrmann, S.D.; Meckes, N.; Bassett, D.R., Jr.; Tudor-Locke, C.; Greer, J.L.; Vezina, J.; Whitt-Glover, M.C.; Leon, A.S. 2011 Compendium of Physical Activities: A second update of codes and MET values. *Med. Sci. Sports Exerc*. **2011**, 43, 1575–1581. https://doi.org/10.1249/MSS.0b013e31821ece12.
